# Gender differences in the impact of illiteracy on dementia risk among community‐dwelling older adults

**DOI:** 10.1002/alz.085830

**Published:** 2025-01-09

**Authors:** Dae Jong Oh, Ji Won Han, Tae Hui Kim, Kyung Phil Kwak, Bong Jo Kim, Shin Gyeom Kim, Jeong Lan Kim, Seok Woo Moon, Joon Hyuk Park, Seung‐Ho Ryu, Jong Chul Youn, Dong Young Lee, Dong Woo Lee, Seok Bum Lee, Jung Jae Lee, Jin Hyeong Jhoo, Ki Woong Kim

**Affiliations:** ^1^ Kangbuk Samsung Hospital, Seoul Korea, Republic of (South); ^2^ Seoul National University Bundang Hospital, Seongnam Korea, Republic of (South); ^3^ Yonsei University Wonju Severance Christian Hospital, Wonju Korea, Republic of (South); ^4^ Dongguk University Gyeongju Hospital, Gyeongju Korea, Republic of (South); ^5^ Gyeongsang National University Hospital, Jinju Korea, Republic of (South); ^6^ Soonchunhyang University Bucheon Hospital, Bucheon Korea, Republic of (South); ^7^ Chungnam National University, Daejeon Korea, Republic of (South); ^8^ School of Medicine, Konkuk University, Konkuk University Chungju Hospital, Chungju Korea, Republic of (South); ^9^ Jeju national University School of Medicine, Jeju‐si Korea, Republic of (South); ^10^ Konkuk University Medical Center, Seoul Korea, Republic of (South); ^11^ Kyunggi Provincial Hospital for the Elderly, Yongin Korea, Republic of (South); ^12^ Seoul National University College of Medicine, Seoul Korea, Republic of (South); ^13^ Inje University Snaggye Paik Hospital, Seoul Korea, Republic of (South); ^14^ Dankook University Hospital, Cheonan Korea, Republic of (South); ^15^ Kangwon National University, School of Medicine, Chuncheon Korea, Republic of (South); ^16^ Seoul National University College of Natural Sciences, Seoul Korea, Republic of (South)

## Abstract

**Background:**

Illiteracy is highly prevalent in older women than in men, but whether the impact of illiteracy on dementia risk is differed by gender remains unclear.

**Method:**

We enrolled 5,217 non‐demented older adults aged ≥ 60 years from the Korean Longitudinal Study on Cognitive Aging and Dementia. Presence of illiteracy was assessed by research nurses and the diagnoses of incident dementia and Alzheimer’s disease (AD) were made by standardized diagnostic interview by geriatric psychiatrists. Neuropsychologists assessed the cognitive performance by using Korean version of Consortium to Establish a Registry for Alzheimer’s Disease Assessment Packet Neuropsychological Assessment Battery. Multivariate Cox proportional hazard regression and linear mixed model analyses were used to identify the impact of illiteracy on the risks of all‐cause dementia/AD and cognitive decline during the 8‐year follow‐up respectively.

**Result:**

Totally, 283 (98 men [4.4%]; 185 women [6.2%]) cases of all‐cause dementia and 227 (71 men [3.2%]; 156 women [5.2%]) cases of AD were identified during the follow‐up period (5.8 ± 2.4 years). Among 901 illiterate participants, 9 (7.9%) men and 101 (12.8%) women developed all‐cause dementia whereas 5 (4.4%) men and 89 (11.3%) women developed AD. Illiteracy was found to pose 2.8‐fold (Hazard Ratio [HR] = 2.79, 95% Confidence Interval [CI] = 1.95 – 3.99) and 2.9‐fold (HR = 2.89, 95% CI = 1.97 – 4.25) increased risks of all‐cause dementia and AD respectively in women. The HRs for association of illiteracy with all‐cause dementia (HR = 2.48, 95% CI = 1.66 – 3.68) and AD (HR = 2.54, 95% CI = 1.66 – 3.90) risks remained significant even when we separately analyzed the less educated women (educational years ≤ 6 years, n = 1,830). However, there was no significant association in men. The impact of illiteracy on the progression of cognitive decline was more pronounced in women (*β* = ‐0.962, SE = 0.147) than in men (*β* = ‐0.618, SE = 0.338).

**Conclusion:**

The impact of illiteracy on dementia risk may be more significant in women than in men, irrespective of the educational attainment of the study population. Illiterate older women should be considered the primary target population for dementia prevention strategies.

